# Metabolic recovery of lipodystrophy, liver steatosis, and pancreatic β cell proliferation after the withdrawal of OSI-906

**DOI:** 10.1038/s41598-017-04304-5

**Published:** 2017-06-23

**Authors:** Kazuki Tajima, Jun Shirakawa, Yu Togashi, Shunsuke Yamazaki, Tomoko Okuyama, Mayu Kyohara, Hiromi Konishi, Yasuo Terauchi

**Affiliations:** Department of Endocrinology and Metabolism, Graduate School of Medicine, Yokohama-City University, Yokohama, Japan 236-0004 Japan

## Abstract

Growth factor signaling via insulin receptor (IR) and IGF-1 receptor (IGF1R) plays several important roles in the pathogenesis of metabolic syndrome and diabetes. OSI-906 (linsitinib), an anti-tumor drug, is an orally bioavailable dual inhibitor of IR and IGF1R. To investigate the recovery from metabolic changes induced by the acute inhibition of IR and IGF1R in adult mice, mice were treated with OSI-906 or a vehicle for 7 days and the results were analyzed on the last day of injection (Day 7) or after 7 or 21 days of withdrawal (Day 14 or Day 28). On day 7, the visceral white fat mass was significantly reduced in mice treated with OSI-906 accompanied by a reduced expression of leptin and an increased expression of the lipolysis-related genes Lpl and Atgl. Interestingly, the lipoatrophy and the observed changes in gene expression were completely reversed on day 14. Similarly, liver steatosis and β cell proliferation were transiently observed on day 7 but had disappeared by day 14. Taken together, these results suggest that this model for the acute inhibition of systemic IR/IGF1R signaling may be useful for investigating the recovery from metabolic disorders induced by impaired growth factor signaling.

## Introduction

Pathological changes in metabolic tissues, such as adipose tissue, liver, and pancreatic β cells, are observed during the development of metabolic disorders^1^. Both adipose tissue expansion and lipodystrophy are associated with insulin resistance-related diseases, such as metabolic syndrome, congenital generalized lipodystrophy, and acquired lipodystrophy including HIV lipodystrophy, cachexia, and autoimmune disorders^2–4^. Fatty liver occurs in patients with components of metabolic syndrome as a result of increased fatty acids uptake arising from impaired insulin action in adipose tissue^5, 6^. Diabetes develops when the compensatory growth of pancreatic β cells is insufficient to produce an appropriate amount of insulin, which can overcome insulin resistance^7^. However, to explore proper therapeutic strategies for patients who already possess abnormal changes in those metabolic tissues, a physiological model for the recovery from insulin resistance-induced metabolic changes in all of these tissues is urgently required.

Growth factor signaling via the insulin receptor (IR) and the IGF-1 receptor (IGF1R) transmits insulin’s actions to metabolic responses in systemic tissues including adipose tissue, liver, and pancreatic islet cells^8, 9^. Tissue-specific IR, IGF1R, or double-knockout models have been developed^9–16^. Both mice with the fat-specific knockout of IR (FIRKO) and mice carrying fat lacking IR and IGF1R (FIGIRKO) exhibit a reduced white fat mass^14, 16^. Liver-specific insulin receptor knockout (LIRKO) mice manifest severe insulin resistance and glucose intolerance, leading to a significant increase in β cell mass as well as the failure of insulin to suppress hepatic glucose production^15^. Mice with the tissue-specific knockout of the insulin receptor in β cells (βIRKO) show defective glucose sensing and a reduced β cell mass^11, 13^, and mice lacking IGF-1 receptors in β cells (βIGFRKO) have defects in glucose-stimulated insulin secretion without any alteration in β cell mass^10, 17^. Compound β cell-specific insulin receptor and IGF-1 receptor knockout (βDKO) mice developed diabetes at 3 weeks after birth, in contrast to the milder phenotypes observed in single mutants^9^. These studies provide genetic evidence of the crucial role for insulin and IGF-1 signaling in regulating β cell mass and function, with a prominent role for insulin.

Since the pathological changes in metabolic tissues are irreversible in genetically engineered models, the mechanisms underlying restoration to normal metabolic conditions have remained unsolved. Furthermore, insulin resistance occurs in not only a specific tissue, but also in various systemic tissues in the pathophysiological state. Because systemic IR-deficient or IGF1R-deficient models are embryonic lethal^18–20^, a model that induces the transient blockade of systemic growth factor signaling might be a useful for analyzing the mechanisms of metabolic tissue restoration.

OSI-906 (linsitinib) is an orally bioavailable dual insulin/IGF-1 receptor tyrosine kinase inhibitor^21^. By inhibiting the autophosphorylation of these receptors, OSI-906 inhibits their downstream pathways, such as the phosphorylation of Akt, ERK1/2, and p70S6K^21^. OSI-906 has been shown, using animal models, to be a promising therapeutic agent for several cancers^22^. A recent report showed that *in vitro* treatment with OSI-906 reduced cellular transformation in BALB/c-3T3 cells in accordance with inhibition of the PI3K-Akt pathway^23^. OSI-906 has also shown preliminary evidence of anti-tumor activity in several solid tumors in phase I clinical trials^24^. We previously showed that the oral administration of OSI-906 not only exacerbated severe glucose intolerance and insulin resistance in a rapid manner, but also increased the β cell mass and β cell proliferation in mice^25^.

In this study, we conducted histological and gene expression analyses of adipose tissue, liver, and pancreatic β cells to assess the recovery from pathological changes in these tissues after the withdrawal of OSI-906 *in vivo*.

## Results

### OSI-906 induced transient hyperglycemia and hyperlipidemia accompanied by hyperinsulinemia

**Figure 1:**
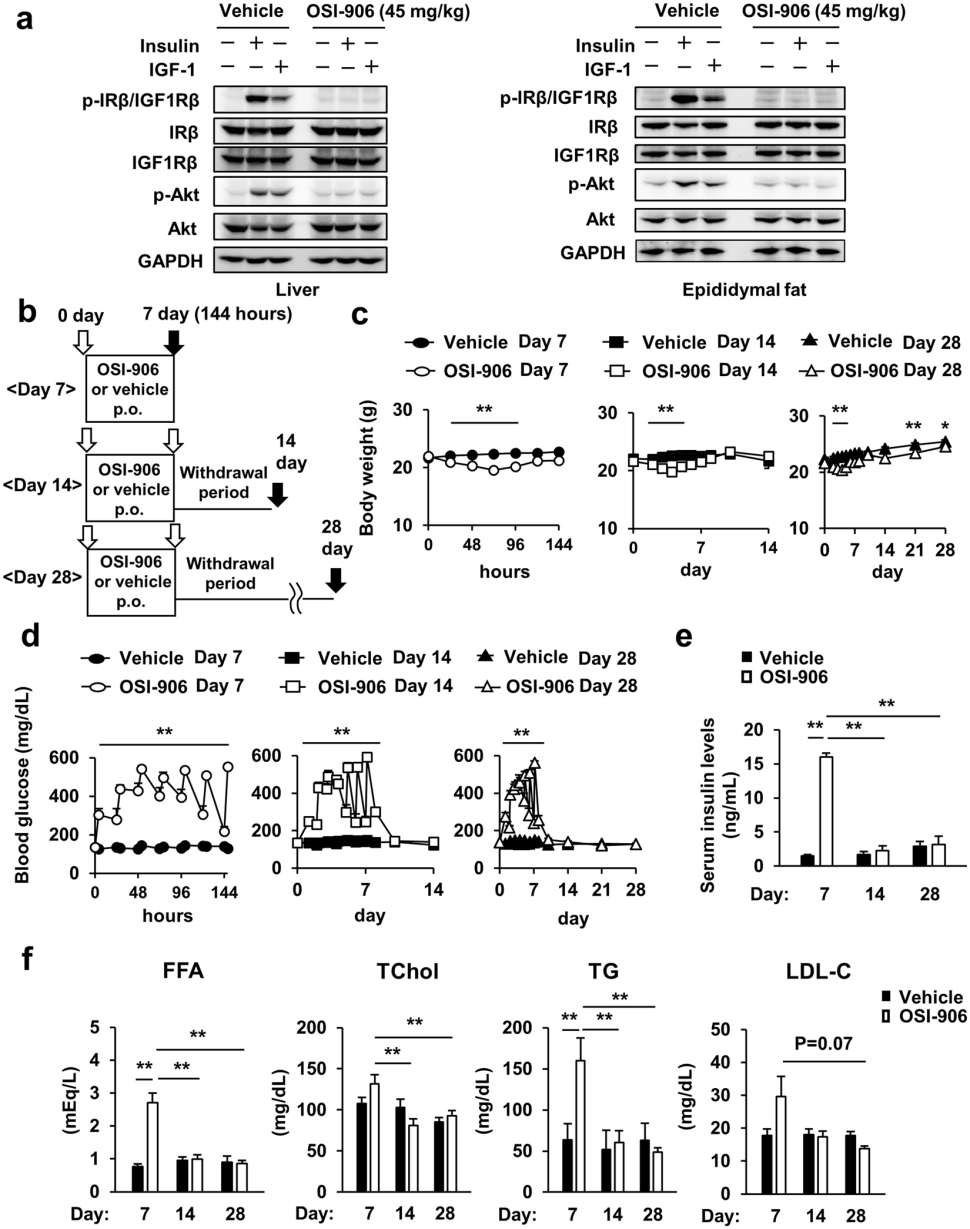
Transient hyperglycemia accompanied by ephemeral hyperinsulinemia induced by IR and IGF1R inhibition with OSI-906. (**a**) C57BL/6J mice were subjected to a 16-hour fast and OSI-906 (45 mg/kg) or a vehicle (Solutol HS-15) was administered orally 1 hour before injection with either saline, 10 units of insulin, or 1 mg/kg of IGF-1 via the inferior vena cava. The liver and epididymal fat were collected 70 and 120 seconds after injection, respectively. The total protein extracts from the liver and epididymal fat were subjected to immunoblotting for p-IRβ/IGF1Rβ, IRβ, IGF1Rβ, p-Akt, Akt, and GAPDH. (**b**) Experimental protocol. (n = 8 per group). (**c**) Body weight gain. Data represent the mean ± SEM. *P ≤ 0.05, **P ≤ 0.01 v.s. OSI-906 (n = 8 per group). (**d**) Blood glucose levels. Data represent the mean ± SEM. **P ≤ 0.01 v.s. OSI-906 (n = 8 per group). (**e**) Serum insulin levels during the fed state on days 7, 14, and 28. Data represent the mean ± SEM. **P ≤ 0.01 (n = 5 per group). (**f**) Plasma free fatty acid (FFA), total cholesterol (TChol), triglyceride (TG), and low-density lipoprotein-cholesterol (LDL-C) levels in both groups on days 7, 14, and 28. Data represent the mean ± SEM. **P ≤ 0.01 (n = 5 per group).

In this study, we administered OSI-906 orally to mice at a dose of 45 mg/kg, which enabled the sufficient inhibition of IR and IGF1R. One hour after OSI-906 treatment, increased blood glucose and serum insulin levels were observed (see Supplementary Fig. S1a,b). The phosphorylation of IR/IGF1R or Akt in the liver or epididymal fat after the injection of either insulin or IGF-1 via the inferior vena cava was completely blunted after the injection of OSI-906 (Fig. 1a). Thus, the administration of this dose of OSI-906 was sufficient to prevent the activities of IR and IGF1R *in vivo*.

We administered OSI-906 orally to mice once daily for 7 days and sacrificed the mice on the day of the last OSI-906 administration (Day 7) or after 7 or 21 days of withdrawal from the OSI-906 treatment (Day 14 or Day 28) (Fig. 1b). OSI-906 elicited a significant body weight loss from day 2 to day 5, but the body weights of the OSI-906-treated mice had returned to normal by days 7 and 14 (Fig. 1c). Food intake and the consumption of drinking water were significantly lower on day 2 but were significantly higher on days 5 to 8 in mice treated with OSI-906, compared with the levels in the control group (see Supplementary Fig. S1c,d). The OSI-906-treated mice showed continuous hyperglycemia and hyperinsulinemia until day 7 (Fig. 1d,e). Immediately after the end of the OSI-906 treatment, the mice started to recover to their normal blood glucose levels, and the hyperinsulinemia completely disappeared within 7 days (Fig. 1d,e). The plasma level of 3-hydroxybutyrate (3-HB), which is the major ketone body, was not significantly increased in the OSI-906-treated mice on day 7 (see Supplementary Fig. S1e). The plasma GPT level was not changed by OSI-906 treatment (see Supplementary Fig. S1f). The plasma level of leptin tended to be decreased in the OSI-906-treated mice on day 7, but the decreased leptin level had returned to the normal level by day 14 (see Supplementary Fig. S1g). The plasma free fatty acid and triglyceride levels were significantly increased on day 7, but these levels abated within a week after the end of OSI-906 treatment (Fig. 1f). The treatment with OSI-906 did not significantly increase the TChol and LDL-C levels on day 7 (Fig. 1f). Collectively, hyperglycemia, hyperinsulinemia, and hyperlipidemia induced by the inhibition of IR and IGF1R improved after the withdrawal of OSI-906 treatment.

### Lipodystrophy, hepatic steatosis and increased β cell proliferation induced by OSI-906 were restored to normal within 7 days after withdrawal

**Figure 2:**
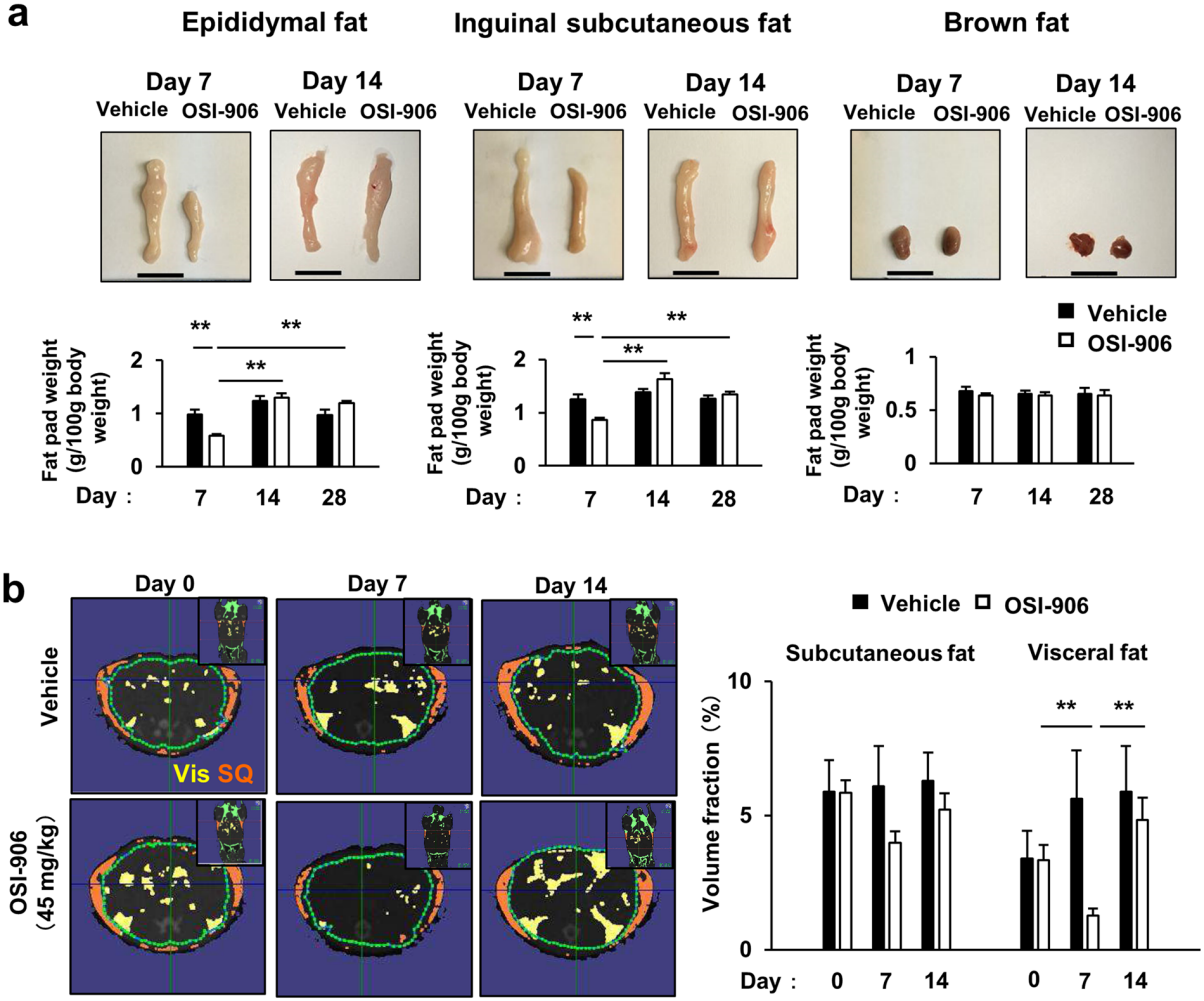
Adipose tissue atrophy accompanying IR and IGF1R inhibition was restored within a week. (**a**) (upper) Representative fat pads of epididymal fat (left), inguinal subcutaneous fat (center), and brown fat (right). (lower) The ratios of the weights of the epididymal fat, inguinal subcutaneous fat, and brown fat relative to the body weights on days 7, 14, and 28. Data represent the mean ± SEM. **P ≤ 0.01 (n = 8 per group). Scale bar = 1 cm. (**b**) (left) Representative computerized tomography sections of abdominal regions of mice on days 0, 7, and 14. Visceral fat is shown in yellow, and subcutaneous fat is shown in orange. Vis, visceral fat; SQ, subcutaneous fat. (right) The volume fraction of subcutaneous and visceral fat mass on days 0, 7, and 14. Data represent the mean ± SEM. **P ≤ 0.01 (n = 3 per group).

**Figure 3:**
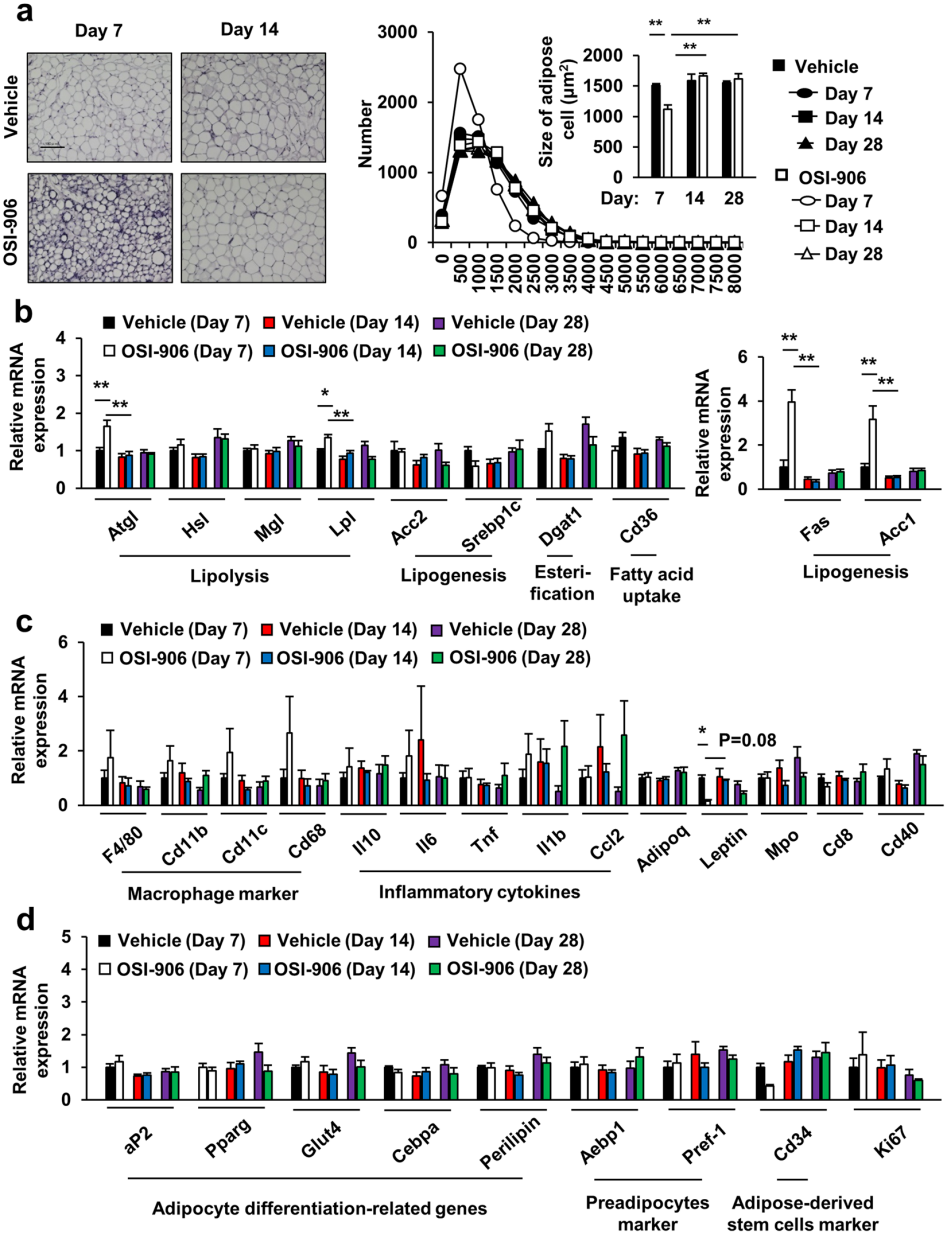
Adipocyte size and gene expressions in epididymal fat. (**a**) (left) Hematoxylin and eosin-stained sections of epididymal fat on days 7 and 14. Scale bar = 100 μm. (right) Histogram of the adipocyte size and mean cell size of the epididymal fat on days 7, 14, and 28. Data represent the mean ± SEM. **P ≤ 0.01 (n = 5 per group). (**b**–**d**) The mRNA expression levels of the indicated molecules in epididymal fat on days 7, 14 and 28. Data represent the mean ± SEM. *P ≤ 0.05, **P ≤ 0.01 (n = 5-6 per group).

We evaluated the effect of OSI-906 on the adipose tissues. OSI-906 significantly decreased the weights of both epididymal and inguinal subcutaneous fat, whereas it did not affect the brown adipose tissue weight (Fig. 2a). The reduction in white adipose tissue weight was rapidly restored to the levels observed in the control mice within 7 days after the last administration of OSI-906 (Fig. 2a). We also evaluated the atrophic changes in adipose tissues using computerized tomography imaging. OSI-906 treatment had induced a significant reduction in the volume fraction of visceral fat on day 7, but the volume fraction was restored to pre-treatment levels by day 14 (Fig. 2b). We next examined the adipocyte size in epididymal fat. OSI-906 treatment had induced a significant reduction in adipocyte size on day 7, but the size was rapidly restored, reaching the same value observed in the control mice by day 14 (Fig. 3a). In contrast, no difference in cell size in the brown adipose tissue was seen between the vehicle-treated and the OSI-906-treated mice (see Supplementary Fig. S2). The expression levels of the lipolysis-related genes Atgl and Lpl, but not of Hsl and Mgl, were significantly upregulated in the epididymal fat of OSI-906-treated mice on day 7, but these increased expressions were reversed to the normal state within a week (Fig. 3b). OSI-906 also transiently upregulated the lipogenic genes Fas and Acc1, but not Srebp1c (Fig. 3b). No significant changes in the expression levels of Cd36 and Dgat1, two key molecules for fatty acid uptake and esterification, respectively, were seen (Fig. 3b). Atrophic visceral fat showed no significant changes in the expressions of macrophage markers, such as F4/80, Cd11b, Cd11c, and Cd68, or in inflammatory cytokines, such as Tnf, Il6, Il1b, and Ccl2 (Fig. 3c). The gene expressions of Mpo, Cd8, and Cd40, which are markers for neutrophils, T cells, and B cells, respectively, were not increased in OSI-906-treated mice on day 7 (Fig. 3c). The serum adiponectin and leptin levels are known to be extremely low in patients with lipodystrophy, as well as in rodent models of lipodystrophy^26, 27^. The expression level of leptin, but not adiponectin, was significantly lower in mice treated with OSI-906 than in the control mice, and leptin expression was rapidly restored to the normal level within a week after the withdrawal of OSI-906 (Fig. 3c). The expression levels of the adipocyte differentiation-related genes aP2, Pparg, Glut4, Cebpa, and Perilipin were unchanged in the OSI-906-treated mice, compared with those in the control mice, on days 7 and 14 (Fig. 3d). Moreover, no significant changes in the expressions of Pref-1, Aebp1, Cd34, and Ki67, which are markers for preadipocytes, adipose-derived stem cells, or proliferation, were seen (Fig. 3d). These results suggested that the inhibition of IR and IGF1R resulted in lipodystrophy in accordance with a reduced expression of leptin and an increased expression of the lipolysis-related genes Lpl and Atgl. The lipodystrophy and the observed changes in gene expression returned to normal levels after the withdrawal of OSI-906.

**Figure 4:**
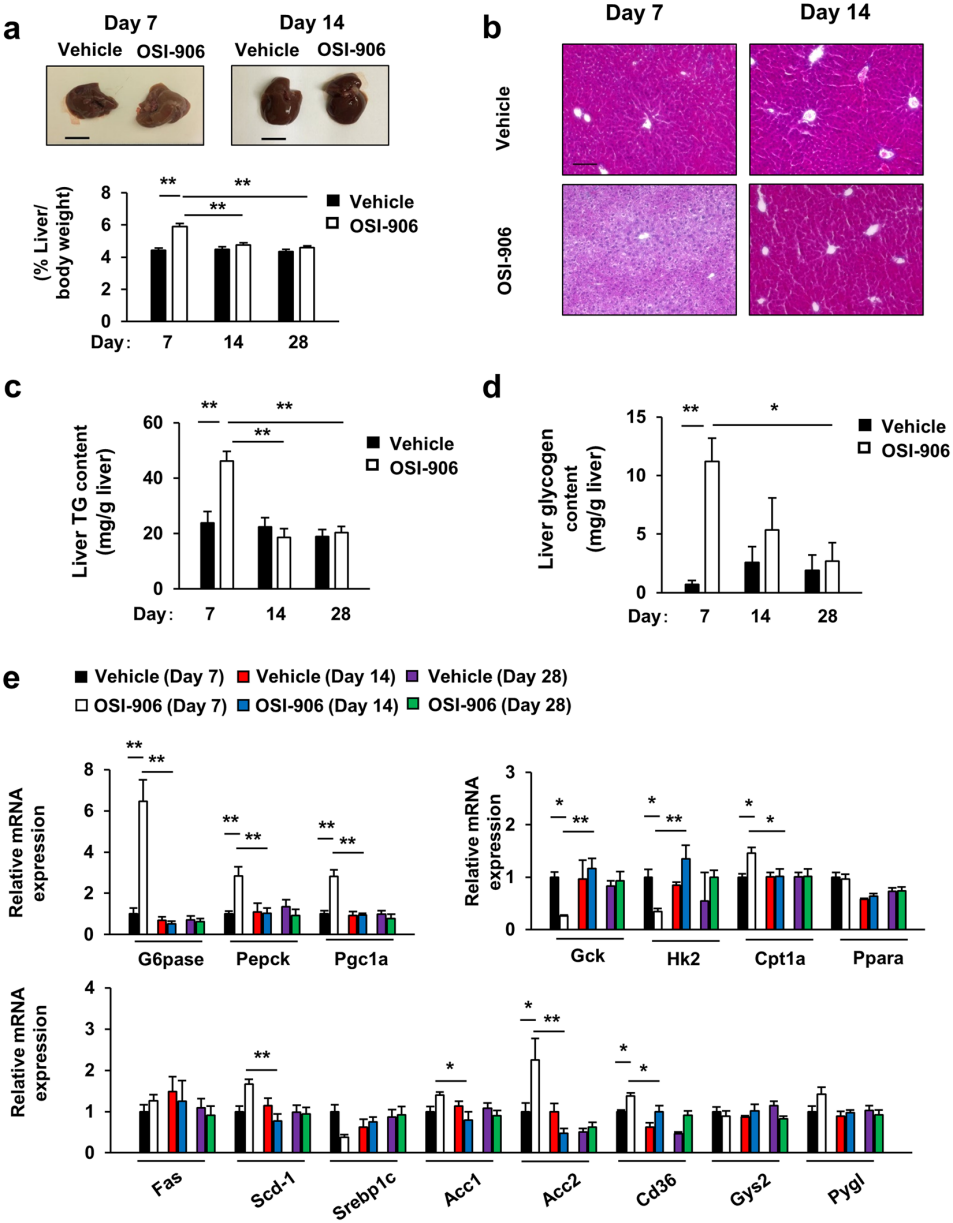
Fatty liver caused by IR and IGF1R inhibition with OSI-906 was restored after washout. (**a**) (lower) Ratios of the liver weights to the body weights on days 7, 14, and 28. Data represent the mean ± SEM. **P ≤ 0.01 (n = 8 per group) (upper) Representative livers. Scale bar = 1 cm. (**b**) Hematoxylin and eosin-stained sections of liver on days 7 and 14. Scale bar = 100 μm. (**c**,**d**) Triglyceride (**c**) and glycogen (**d**) contents in the liver on days 7, 14, and 28. Data represent the mean ± SEM. *P ≤ 0.05, **P ≤ 0.01 (n = 4 per group). (**e**) mRNA expression levels of the indicated molecules in the liver on days 7, 14, and 28. Data represent the mean ± SEM. *P ≤ 0.05, **P ≤ 0.01 (n = 6 per group).

We next examined the effect of OSI-906 on the liver. The administration of OSI-906 enlarged the liver size, accompanied by an increased liver weight, and had induced hepatic steatosis by day 7; however, these values were restored to the levels observed in the control mice by day 14 (Fig. 4a,b). These histological changes were consistent with the transient increases in hepatic triglyceride contents and glycogen contents observed on day 7 (Fig. 4c,d). Apparent histological inflammatory changes were not detected in these short-term experiments (Fig. 4b). The expression levels of gluconeogenic genes, such as glucose-6-phosphatase (G6pase) and phosphoenolpyruvate carboxykinase (Pepck), were significantly increased in the livers of mice treated with OSI-906 on day 7, whereas the expression of glucokinase (Gck) and hexokinase 2 (Hk2), which are genes encoding glycolytic enzymes, were decreased (Fig. 4e). The expressions of Pgc1a and Cpt1a were also augmented by OSI-906 treatment (Fig. 4e). All of these changes in the gene expression levels were restored to the levels observed in the control mice within a week after the withdrawal of OSI-906 (Fig. 4e). The expressions of Ppara and genes related to de novo lipogenesis, such as Fas, Scd-1, Srebp1c, and Acc1, were not altered (Fig. 4e). In contrast, the expression of Cd36, a fatty acids transporter, was significantly increased in the liver of mice treated with OSI-906 on day 7, but the increased expression had returned to the normal level by day 14, as reflected by the amelioration of steatosis (Fig. 4e). The expressions of liver glycogen synthase-2 (Gys2), a key enzyme in glycogen synthesis, and Pygl, an effector regulating glycogenolysis, were not changed (Fig. 4e). These results suggested that the rapid induction of fatty liver induced by IR and IGF1R inhibition was, at least in part, caused by the accumulation of triglycerides in the liver as a result of the increased CD36-mediated uptake of fatty acids.

**Figure 5:**
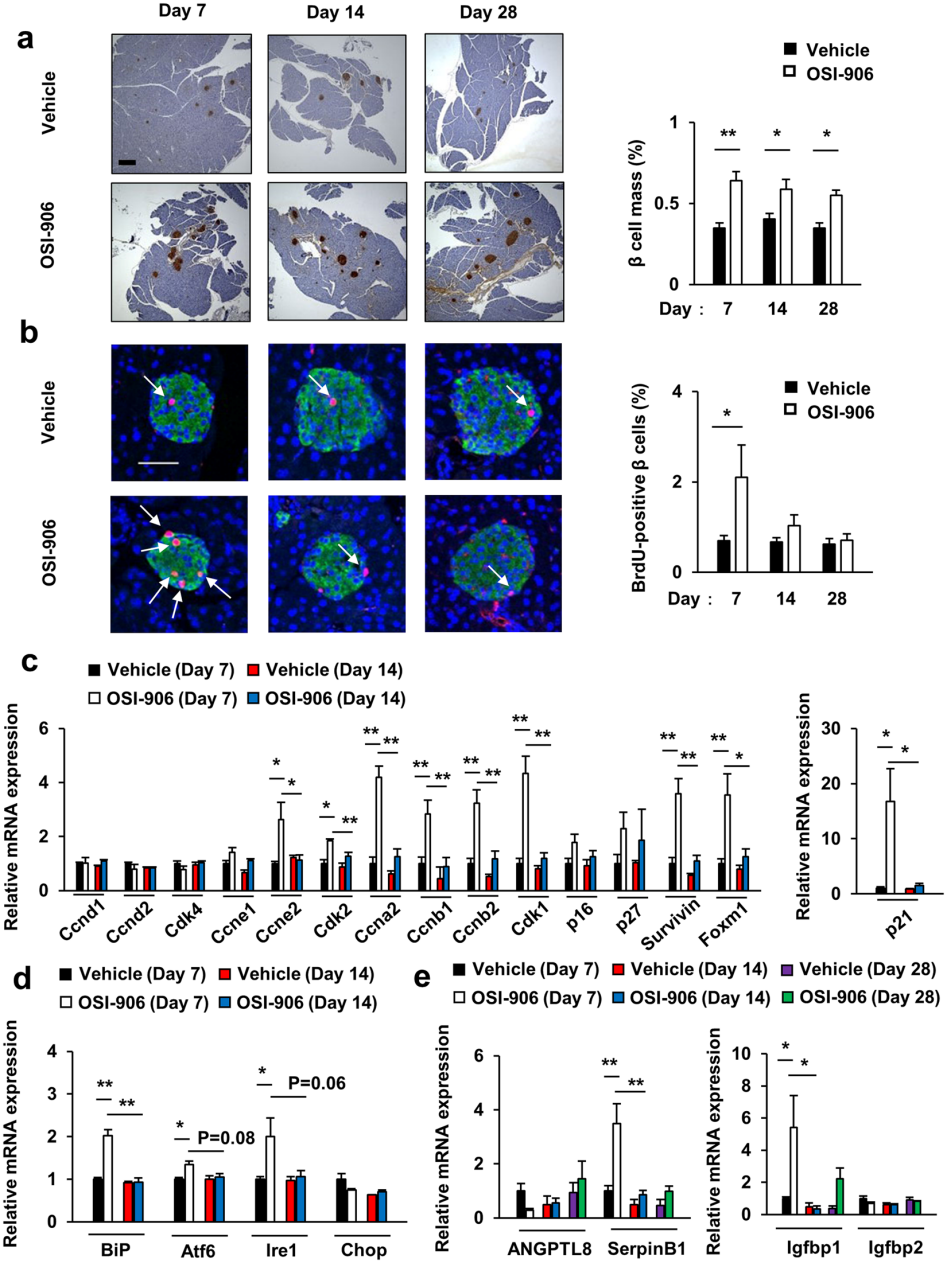
Transient β cell proliferation induced by peripheral IR and IGF1R inhibition with OSI-906. (**a**) (left) Pancreatic sections were stained with antibodies to insulin. The scale bar represents 300 μm. (right) β Cell mass with β cell area shown as the percent area of the entire pancreas. Data represent the mean ± SEM. *P ≤ 0.05, **P ≤ 0.01 (n = 7 per group). (**b**) (left) Pancreatic sections were stained with antibodies to DAPI (blue), insulin (green), and BrdU (red). Scale bar = 50 μm. (right) Number of BrdU-positive β cells in the islets (at least 50 islets per indicated group). Data represent the mean ± SEM. *P ≤ 0.05 (n = 5 per group). (**c**–**e**) mRNA expression levels of the indicated molecules in the islets (n = 4 per group) (**c**,**d**) and in the liver (n = 3 per group) (**e**). Data represent the mean ± SEM. *P ≤ 0.05, **P ≤ 0.01.

Pancreatic β cell proliferation is observed in response to peripheral insulin resistance in mice through the activation of insulin signaling in β cells^13, 28^. We previously reported that the once daily administration of 40 mg/kg of OSI-906 for 7 days significantly increased β cell proliferation and β cell mass^25^. Similarly, OSI-906 had significantly increased the β cell mass and β cell proliferation by day 7 in the present study (Fig. 5a,b). No changes in the weight of the pancreas were observed between the vehicle-treated and the OSI-906-treated mice (see Supplementary Fig. S3a). Interestingly, the increased β cell proliferation had been restored to the levels observed in the control mice at 7 days after the last administration of OSI-906, although the increased β cell mass remained larger than that observed in the vehicle-treated control group (Fig. 5a,b). In islets from mice treated with OSI-906, a marked upregulation of Ccna2, Ccnb1, Ccnb2, Ccne2, Cdk1, Cdk2, Survivin, Foxm1, and p21 was observed on day 7, compared with the levels observed in islets from control mice, whereas the expressions of Ccnd1, Ccnd2, Cdk4, p16, and p27 were unchanged (Fig. 5c). The administration of OSI-906 also did not upregulate Cyclin D2 immunofluorescence on day 7 (see Supplementary Fig. S3b). All of the upregulated genes involved in the cell cycle were restored to their normal expression levels within a week after the withdrawal of OSI-906 (Fig. 5c). The expression levels of the ER stress-related molecules BiP, Atf6, and Ire1 in the islets from OSI-906-treated mice were significantly upregulated on day 7, whereas the expression of Chop was unchanged (Fig. 5d). The expression of Igfbp1, but not Igfbp2, in the liver was significantly augmented by OSI-906 (Fig. 5e), suggesting a state of hepatic insulin resistance with the efficient inhibition of insulin action^29^. ANGPTL8/βtrophin and SerpinB1 are potential candidates for liver-derived circulating factors that promote β cell proliferation under insulin resistance^30, 31^. The expression level of SerpinB1 in the liver was significantly increased on day 7 after the start of OSI-906 administration, whereas that of ANGPTL8/βtrophin remained unchanged (Fig. 5e).

### Leptin supplementation reduced food intake and improved hepatic lipid accumulation in OSI-906-treated mice

**Figure 6:**
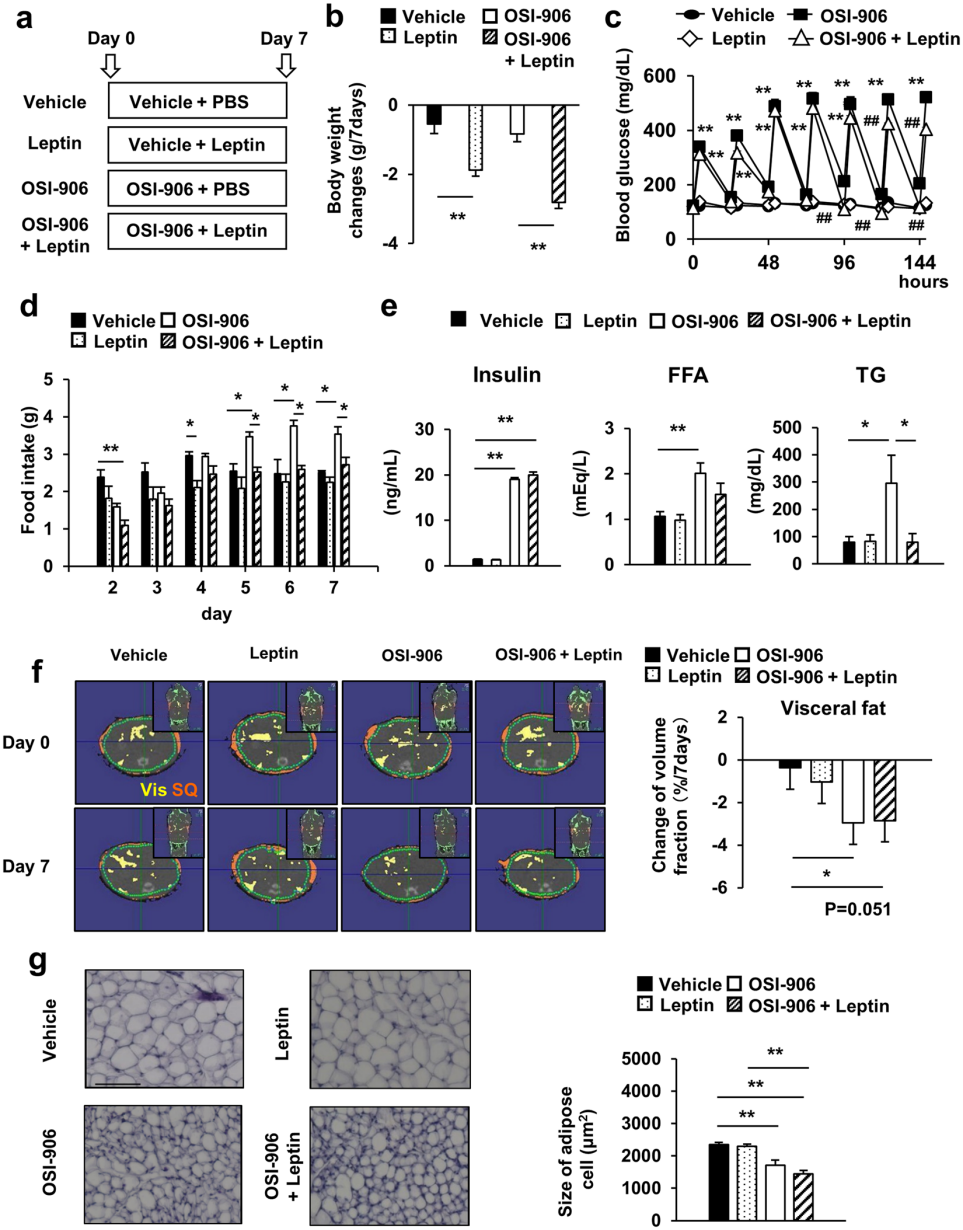
Leptin treatment reduced food intake and body weight gain in OSI-906-treated mice. (**a**) Experimental protocol. OSI-906 (45 mg/kg) or the vehicle was administered orally once daily (at 7:00), and leptin (0.5 mg/g) or PBS was subcutaneously injected twice daily (at 7:00 and 19:00) for seven days. The experiments were performed in 8-week-old C57BL/6J mice (n = 9–10 per group). (**b**) Body weight changes from day 0 to day 7. Data represent the mean ± SEM. *P ≤ 0.05, **P ≤ 0.01 (n = 9–10 per group). (**c**) Blood glucose levels just before and 4 hours after the administration of OSI-906 or the vehicle. Data represent the mean ± SEM. (n = 9–10 per group). **P ≤ 0.01 vs. Vehicle. ^##^ *P* ≤ 0.01 vs. OSI-906. (**d**) Daily food intake. Mice were housed 3–4 per cage. Food intake were monitored per cage during a period of 7 days. Data represent the mean ± SEM. *P ≤ 0.05, **P ≤ 0.01. (**e**) Serum insulin (n = 6 per group), FFA, and TG (n = 9–10 per group) during the fed state on days 7. Data represent the mean ± SEM. *P ≤ 0.05, **P ≤ 0.01. (**f**) (left) Representative computerized tomography sections of abdominal regions of mice on days 0 and 7. Visceral fat is shown in yellow, and subcutaneous fat is shown in orange. Vis, visceral fat; SQ, subcutaneous fat. (right) The change of volume fraction of visceral fat mass from days 0 to day 7. Data represent the mean ± SEM. *P ≤ 0.05 (n = 6 per group). (**g**) (left) Hematoxylin and eosin-stained sections of epididymal fat on days 7. Scale bar = 100 μm. (right) Mean cell size of the epididymal fat on days 7. Data represent the mean ± SEM. **P ≤ 0.01 (n = 6 per group).

Generalized lipodystrophy in humans is attributed to a deficiency of leptin^26^. Human and rodent studies have shown that leptin treatment rescues insulin resistance, hepatic steatosis and diabetes^27, 32^. To evaluate the effect of leptin administration in OSI-906-treated mice, we treated mice with OSI-906 in combination with subcutaneous leptin injections for 7 days (Fig. 6a). The body weight change over the course of 7 days in both OSI-906 and leptin-treated mice was comparable to that in control or OSI-906-treated mice (Fig. 6b). Leptin supplementation improved hyperglycemia on days 5 to 7 and reduced food intake and water consumption in OSI-906-treated mice (Fig. 6c,d and see Supplementary Fig. S4). Treatment with leptin significantly reduced the serum triglyceride level, but not the levels of insulin or free fatty acids in OSI-906-treated mice (Fig. 6e).

**Figure 7:**
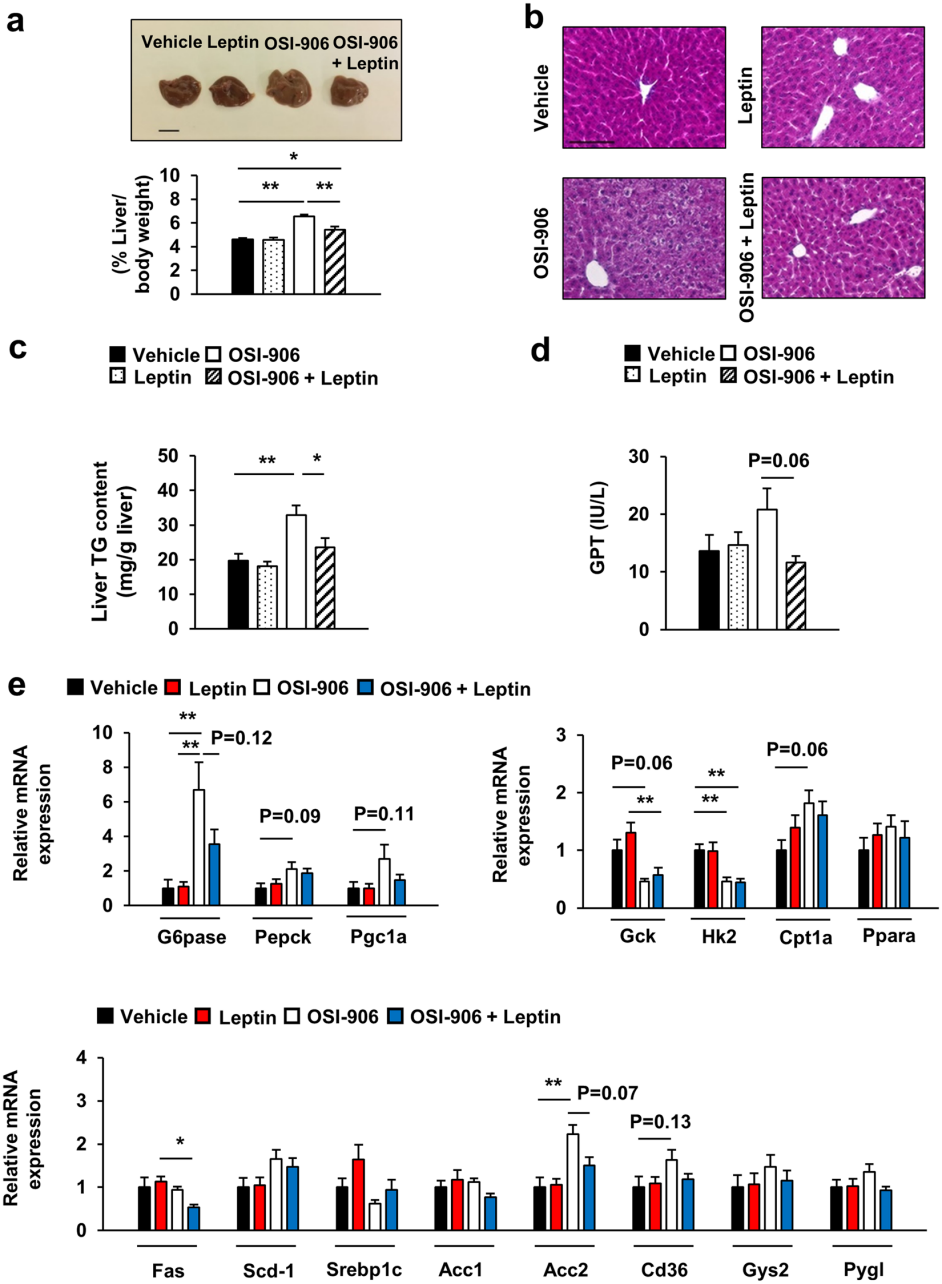
Leptin treatment improved hepatic lipid accumulation induced by IR and IGF1R inhibition with OSI-906. (**a**) (lower) Ratios of the liver weights to the body weights on days 7. Data represent the mean ± SEM. *P ≤ 0.05, **P ≤ 0.01 (n = 6 per group) (upper) Representative livers. Scale bar = 1 cm. (**b**) Hematoxylin and eosin-stained sections of liver on days 7. Scale bar = 100 μm. (**c**) Triglyceride content in the liver on days 7. Data represent the mean ± SEM. *P ≤ 0.05, **P ≤ 0.01 (n = 6 per group). (**d**) Plasma GPT level on days 7. Data represent the mean ± SEM. (n = 9–10 per group). (**e**) mRNA expression levels of the indicated molecules in the liver on days 7. Data represent the mean ± SEM. *P ≤ 0.05, **P ≤ 0.01 (n = 6 per group).

**Figure 8:**
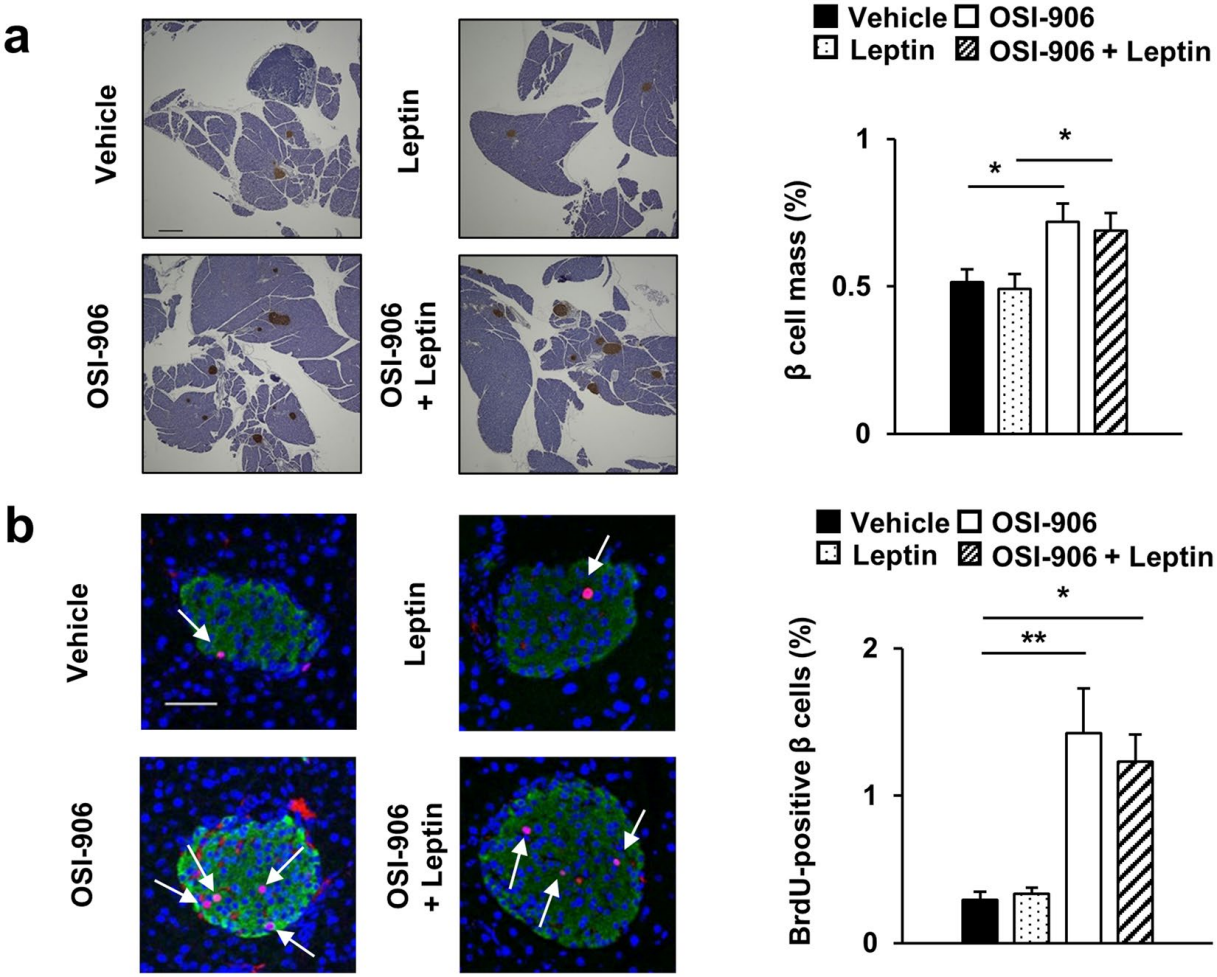
Leptin replacement did not affect pancreatic β cell mass and proliferation in OSI-906-treated mice. (**a**) Pancreatic sections were stained with antibodies to insulin. The scale bar represents 300 μm. β Cell mass with β cell area shown as the percent area of the entire pancreas. Data represent the mean ± SEM. *P ≤ 0.05 (n = 6 per group). (**b**) Pancreatic sections were stained with antibodies to DAPI (blue), insulin (green), and BrdU (red). Scale bar = 50 μm. Number of BrdU-positive β cells in the islets (at least 50 islets per indicated group). Data represent the mean ± SEM. *P ≤ 0.05, **P ≤ 0.01 (n = 6 per group).

We next examined the effect of leptin treatment on adipose tissue, liver and pancreatic β cells. Leptin treatment did not affect the reduced volume fraction of visceral fat or the adipocyte size in OSI-906-treated mice (Fig. 6f,g). Leptin significantly reduced the weight and triglyceride content in the liver and improved steatosis in OSI-906-treated mice (Fig. 7a–c). The plasma GPT level tended to be decreased by treatment with leptin in the OSI-906-treated mice (Fig. 7d). The gene expression analysis in the liver showed that leptin treatment had no significant effects on the gene expression levels in OSI-906-treated mice (Fig. 7e). Leptin replacement also had a minimal effect on β cell mass and proliferation in OSI-906-treated mice (Fig. 8a,b).

## Discussion

Here, we show that mice with IR and IGF1R-specific dual inhibition induced by OSI-906 at 45 mg/kg daily for 7 days developed lipodystrophy, steatosis, and β cell proliferation accompanied by hyperglycemic-hyperinsulinemia and hyperlipidemia. Furthermore, after the washout of the IR and IGF1R inhibitor, adipose tissue atrophy, fatty liver, and β cell outgrowth were reversed to the normal state within 1 week.

In a previous study, mice treated with OSI-906 at 75 mg/kg showed significantly increased blood glucose levels, while treatment with 25 mg/kg of OSI-906 did not induce hyperglycemia^21^. We previously showed that the administration of OSI-906 at 50 or 75 mg/kg not only induced hyperglycemia and hyperinsulinemia but also reduced the survival rate of mice^25^. In contrast, OSI-906 at 40 mg/kg once daily for 7 days elicited sustained hyperglycemia, but not a low survival rate^25^. In this study, we used 45 mg/kg of OSI-906 for 7 days, and no adverse effects on the survival rate were seen.

OSI-906 has been shown to block the ligand-induced activation of downstream pathways, including the phosphorylation of Akt, Erk1/2, and p70S6K, in the 3T3/huIGF-1R cell line (LISN)^21^. The inhibition of IR/IGF-1R signaling by OSI-906 in the two-stage BALB/c-3T3 cell transformation assay (BALB-CTA) diminished the phosphorylation of Akt, p70S6K, S6 protein, 4EBP-1, GSK3β, and AMPK, but not the phosphorylation of Erk1/2^23^. OSI-906 administration at a single dose of 75 mg/kg in the LISN xenograft mouse model inhibited the phosphorylation of IGF-1R for 24 hours^21^. In this study, the insulin- or IGF-1-stimulated phosphorylation of Akt in the liver or epididymal fat was completely blocked one hour after the administration of OSI-906 at 45 mg/kg.

The increased expression of Atgl and Lpl, which are regulatory genes of lipolysis, in OSI-906 treated adipose tissue indicates that the lipodystrophy in our model may be mediated by an impairment in the IR-induced inhibition of lipolysis. The brown adipose tissue showed no significant histological changes after the inhibition of IR and IGF1R for 1 week, although FIGIRKO mice exhibited an almost complete absence of brown adipose tissue^14^. Conditional IGF1R inactivation was previously shown to result in increased white adipose tissue mass and did not affect the brown adipose tissue mass or activity^33^. Therefore, although the inhibition of IR/IGF1R induced by OSI-906 in this study was a nondevelopmental and noncontinuous state, these results suggest that the balance of the degree of inhibition between IR and IGF1R may coordinately regulate the white and brown adipose tissue masses.

Fat atrophy was almost completely recovered by day 14, i.e., 7 days after the withdrawal of OSI-906. No changes in the expressions of genes involved in adipocyte differentiation, proliferation, preadipocytes, and adipose-derived stem cells were observed between day 7 and day 14. Further precise chronological study, especially at an earlier stage after the discontinuation of OSI-906, is required to investigate the mechanisms underlying this rapid recovery of fat mass.

Liver-specific IR knockout (LIRKO) produced severe insulin resistance and hepatic dysplastic lesions, but not steatosis, in the liver^15^. β Cell-specific glucokinase-deficient mice, a model of severely impaired insulin secretion, showed hepatic steatosis and a significant increase in the liver triglyceride content^34^. Thus, the development of fatty liver in this study likely occurred through the action of insulin on non-hepatic tissues, but not on the liver. The overproduction of fatty acids in adipose tissues due to increased lipolysis is the most likely explanation for excess triglyceride accumulation in fatty liver or NAFLD^35, 36^. In fact, the upregulation of Cd36 (a key fatty acids transporter) expression was detected in OSI-906-treated mice. Interestingly, liver steatosis was ameliorated after the withdrawal of OSI-906, which coincided with the recovery of lipodystrophy. Hence, this model may be valuable for exploring the inter-organ network between the liver and adipose tissues in the onset or remission of hepatic steatosis.

We previously showed that glucose signaling regulates ER stress via a mechanism that is partly independent of insulin signaling in β cells^37^. Recently, the β cell unfolded protein response has been reported to regulate murine and human β cell proliferation^38^. Indeed, the expressions of Atf6, Bip, and Ire1 were elevated in proliferating islets from OSI-906-treated mice. Taken together, these results suggest that sustained and continuous adaptive proliferation may exhaust β cells and lead to β cell apoptosis through ER stress or other stress pathways. The transient β cell replication induced by OSI-906 is similar to the situations encountered during pregnancy or a partial pancreatectomy^39, 40^. Indeed, the anti-apoptotic gene Survivin/Birc5, which is essential for β cell mass expansion during pregnancy^40^, is also upregulated in the islets of OSI-906 treated mice. Furthermore, the increased expressions of Cyclin A2, Cyclin B1, FoxM1, and Survivin are quite similar to the gene expression patterns observed after a partial pancreatectomy^39^. Notably, the expression level of Cyclin D2 did not change in mice treated with OSI-906. Since Cyclin D2 is essential for compensatory β cell proliferation downstream of insulin signaling^41^, this result suggests that an insulin signaling-independent pathway contributed to the β cell proliferation observed in this study. This finding is in close agreement with our previous report that in the islets, the tyrosine phosphorylation of the insulin receptor and β cell proliferation induced by glucokinase activation were not affected by OSI-906 treatment^25^. SerpinB1, a protease inhibitor and a liver-derived secretory protein, promotes pancreatic β cell proliferation^31^. Of note, the upregulation of SerpinB1 in the liver of OSI-906-treated mice corresponded to β cell proliferation. Intriguingly, the rapid increase in β cell mass in the OSI-906-treated mice was still larger than that in the vehicle-treated mice even at 21 days after the withdrawal of OSI-906. In contrast, the increased β cell mass during pregnancy is reversed to the levels observed during the pre-pregnant status within ten days after parturition through a decrease in proliferation and an increase in β cell apoptosis^42^. Since the mechanism underlying the decrease in β cell mass through the sensing of ambient conditions remains unknown, the investigation of differences in adaptive β cell reduction between pregnancy and the IR/IGF1R blocking model is an attractive strategy for protecting β cells.

The expression of leptin, but not adiponectin, in fat was significantly lower in the OSI-906-treated mice, but the expression recovered to the normal levels promptly. Leptin replacement reduced blood glucose levels and hepatic steatosis but had no effects on β cell proliferation and lipodystrophy in OSI-906-treated mice. Leptin treatment reduced body weight and improved hyperphagia in OSI-906-treated mice. Circulating leptin and adiponectin levels are reduced in both patients with lipoatrophic diabetes and rodent models of lipodystrophy^43, 44^. Treatment with leptin rescued insulin resistance, hepatic steatosis, and diabetes in both mice and humans with lipodystrophy^27, 32, 45^. Leptin signaling is also involved in β cell replication^46, 47^. Reductions in leptin and insulin signaling in the brain might also affect the changes in energy metabolism and food intake observed in OSI-906-treated mice^48^. These results suggested that the effects of leptin are attributed, in part, to a secondary reduction in food intake, and leptin receptor-mediated signals might be influenced by treatment with OSI-906.

In summary, we established a novel rapid, reversible model for investigating the roles of IR and IGF1R signaling in the maintenance of adipose tissue, liver, and β cells. The results of the present study also show the impact of IR and IGF1R, including the recovery from pathological changes on multiple metabolic tissues, in the context of the therapeutic targeting of IR/IGF1R.

## Methods

### Animals and animal care

C57BL/6J male mice aged 7 weeks were purchased from CLEA Japan. All the mice were fed standard chow (Oriental Yeast, Tokyo, Japan) and were allowed free access to food and water. Body weight was measured just before the administration of OSI-906 or the vehicle until day 7 or on days 8, 10, 14, 21, and 28. All the animal procedures were performed in accordance with the institutional animal care guidelines and the guidelines of the Animal Care Committee of Yokohama City University. The protocol was approved by the Yokohama City University Institutional Animal Care and Use Committee (IACUC) (Permit Number: F-A-13-043). The animal housing rooms were maintained at a constant room temperature (25 °C) under a 12-h light (7:00 A.M.)/dark (7:00 P.M.) cycle.

### Drug treatments

OSI-906 (linsitinib) was purchased from Selleck Chemicals (München, Deutschland), Chemscene (NJ, USA), and MedChem Express (NJ, USA). For the administration experiments, 8-week-old mice were orally treated with 10 μL/g of either the vehicle (30% Solutol HS-15; BASF, Ludwigshafen am Rhein, Germany) or OSI-906 (45 mg/kg) for 7 days between 7:00 P.M. and 8:00 P.M. Solutol HS-15 was dissolved in water at 30% w/v. The powder of OSI-906 was dissolved in 30% Solutol at a concentration of 4.5 mg/mL. The mice were sacrificed after 7 days of daily administration (OSI-906 or vehicle, oral gavage) or after a 7-day or 21-day withdrawal period following 7 days of administration. For the leptin injection experiments, 8-week-old mice treated with or without OSI-906 (45 mg/kg) were subcutaneously injected with PBS or leptin (Peprotech, NJ, USA) (0.5 mg/kg) twice a day for 7 days.

### *In vivo* insulin or IGF-1 stimulation experiments

Mice that had fasted for 16 hours were intraperitoneally anesthetized with 1 mg/kg of pentobarbital and were injected with saline, 10 units of insulin (Humulin; Eli Lilly, Kobe, Japan), or 1 mg/kg of IGF-1 (PeproTech) via the inferior vena cava. The liver and epididymal fat tissues were then collected at 70 and 120 seconds after injection, respectively.

### Measurements of biochemical parameters

The blood glucose levels, serum insulin levels, triglyceride (TG) content in the liver, and glycogen content in the liver were determined using a Glutest Neo Super (Sanwa Chemical Co., Tokyo, Japan), an insulin kit (Morinaga, Tokyo, Japan), a leptin kit (Morinaga) and the Determiner-L TG II kit and the Determiner-GL-E kit (Wako Pure Chemical Industries, Osaka, Japan), respectively. The plasma alanine aminotransferase, free fatty acids, total cholesterol, LDL-cholesterol, and triglyceride levels were assayed using enzymatic methods (Wako Pure Chemical Industries). The plasma 3-hydroxybutyrate level was determined using the β Hydroxybutyrate Assay kit (Abcam, Cambridge, England). The blood glucose levels were checked just before and 4 hours after the administration of OSI-906 or the vehicle until day 7 or on days 8, 10, 14, 21, and 28. Blood samples were taken from the inferior vena cava immediately after sacrifice.

### Histological analysis

The mice were intraperitoneally injected with bromodeoxyuridine (BrdU) (100 mg/kg; Nacalai Tesque, Inc., Kyoto, Japan). More than 5 pancreatic tissue sections separated by 200 μm from each animal were analyzed after fixation and paraffin embedding. The sections were immunostained with antibodies to insulin (Santa Cruz, TX, USA) or BrdU (Dako, Tokyo, Japan). Biotinylated secondary antibodies, a Vectastain elite ABC kit, and a diaminobenzidine substrate kit (Vector, CA, USA) were used to examine the sections using bright-field microscopy to determine the β cell mass, and Alexa Fluor 488- and 555-conjugated secondary antibodies (Invitrogen, CA, USA) were used for the fluorescence microscopy. All the images were acquired using a BZ-9000 microscope (Keyence, Osaka, Japan) or a Fluo View FV1000-D confocal laser-scanning microscope (Olympus, Tokyo, Japan). The percent area of the pancreatic tissue occupied by the β cells was calculated using BIOREVO software (Keyence), as described previously^49^. In the BrdU staining experiment, approximately 50 islets were analyzed using the WinROOF software (Mitani Corp., Tokyo, Japan) to assess the proportion of immunostained nuclei among the insulin-positive cells in each mouse.

Liver and adipose tissue samples were formalin-fixed, embedded in paraffin, sectioned, and stained with hematoxylin and eosin. The white adipocyte areas were measured for 1000 or more cells per mouse in each of the groups using BIOREVO software.

### Immunoblot analysis

The proteins in liver and adipose tissue samples were extracted using T-PER Tissue Protein Extraction Reagent (with proteases and phosphatase inhibitors) (Thermo Scientific, Waltham, MA, USA), as previously described^50^. The extracts were then subjected to immunoblotting with antibodies to p-IR (Tyr1150/1151)/IGF1R (Tyr1135/1136), IR, IGF1R, p-Akt, Akt (all from Cell Signaling Technology, Danvers, MA, USA) and GAPDH (Abcam, MA, USA). Densitometry was performed using Multi gauge V3.0 software (Fuji Film Life Science, Tokyo, Japan).

### Real-time PCR

Total RNA was isolated from pancreatic islets, epididymal fat, and liver using a ribonuclease-free deoxyribonuclease, an RNeasy kit, and a QIA shredder (QIAGEN, Hilden, Deutschland). cDNA was prepared using the High Capacity cDNA reverse transcription kit (Applied Biosystems, CA, USA) and was subjected to quantitative PCR using the THUNDERBIRD SYBR qPCR Mix (Toyobo Co., Ltd., Osaka, Japan). The data were normalized to the mRNA expression levels of β-actin and TATA box-binding protein (Tbp). The real-time PCR primer sequences are listed in see Supplementary Table S1.

### Quantification of subcutaneous and visceral fat using computed tomography

Isoflurane-anesthetized wild-type mice on day 0, day 7, and day 14 were used for a computed tomography (CT) analysis using a cone-beam micro-CT apparatus (R-mCT2; Rigaku, Tokyo, Japan) under the conditions of an FOV of 60 (φ 60 mm × H 60 mm), a 90-kV tube voltage, a 160-μA tube current, and 512 projections/360 degrees. The exposure time was 17 seconds. The quantification of subcutaneous and visceral fat was performed for the area between the proximal end of lumbar vertebra L1 and the distal end of L6 using Fat analysis software (Rigaku), according to the manufacturer’s instructions.

### Statistical analyses

All the data were expressed as the means ± SE and were tested for a normal distribution using JMP software. Statistical differences were calculated according to the Student *t*-test or a one-way ANOVA with an additional Tukey-Kramer and Games-Howell post-hoc test, depending on the homogeneity of the variances. Differences with *P* values < 0.05 (*) or <0.01 (**) were considered significant.

## Electronic supplementary material

Supplementary information
