# Supplementary material for: Metabolic recovery of lipodystrophy, liver steatosis, and pancreatic β cell proliferation after the withdrawal of OSI-906
Source: Sci Rep. 2017 Jun 23;7:4119. doi: 10.1038/s41598-017-04304-5 (PMC5482874; doi:10.1038/s41598-017-04304-5)
Supplement: Supplementary file 1 — Supplementary information [file 41598_2017_4304_MOESM1_ESM.pdf]

Supplementary information:

Supplementary Figure S1-S5
Supplementary Table S1

Metabolic recovery of lipodystrophy, liver steatosis, and pancreatic β cell proliferation after the withdrawal of OSI-906

Kazuki Tajima, Jun Shirakawa, Yu Togashi, Shunsuke Yamazaki, Tomoko Okuyama, Mayu Kyohara, Hiromi Konishi, and Yasuo Terauchi

Supplementary Figure S1

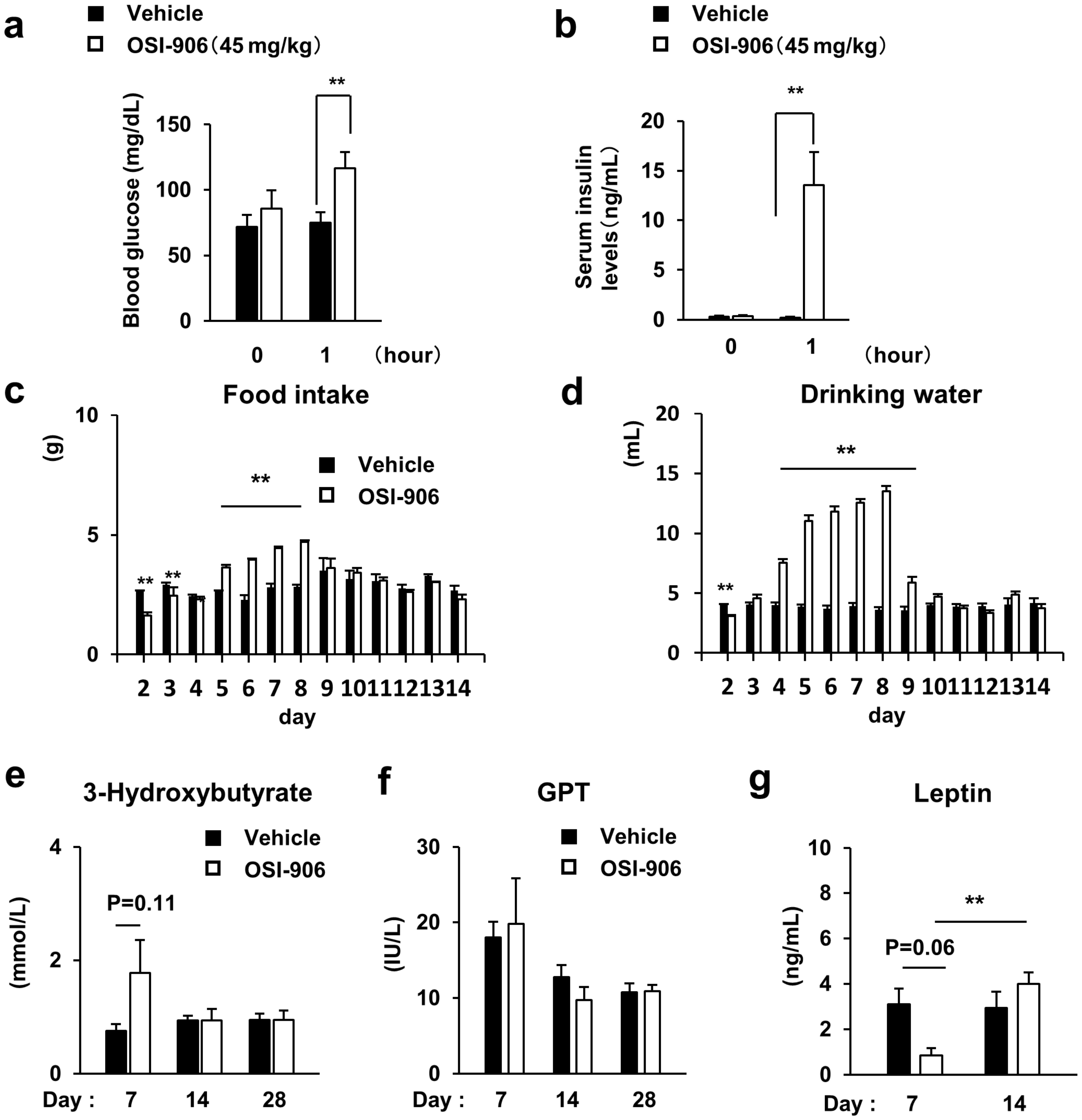

Supplementary Figure S1. The effects of OSI-906 on the metabolic parameters.

(a) and (b): Blood glucose levels (a) and serum insulin levels (b) before and 1 hour after the administration of OSI-906 or the vehicle. Data represent the mean ± SEM. $**P \leq 0.01$ (n = 4 per group). (c) and (d): Daily food intake (c) and consumption of drinking water (d). Mice were housed 4 per cage. Food intake and consumption of drinking water were monitored per cage during a period of 14 days and the results are shown as the mean ± SE. $**P \leq 0.01$. (e) Plasma 3-Hydroxybutyric acid (3-HB) level in both groups on days 7, 14, and 28. Data represent the mean ± SEM. (n = 5 per group). (f) Plasma GPT level in both groups on days 7, 14, and 28. Data represent the mean ± SEM. (n = 5 per group). (g) Plasma leptin level in both groups on days 7 and 14. Data represent the mean ± SEM. $**P \leq 0.01$ (n = 5 per group).

Supplementary Figure S2

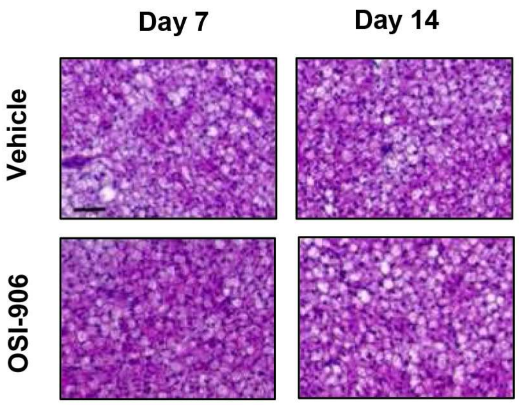

Supplementary Figure S2. OSI-906 had no effects on the morphology of brown adipose tissue. Hematoxylin and eosin-stained sections of brown adipose tissue obtained on days 7 and 14. Scale bar = 50 μm.

Supplementary Figure S3

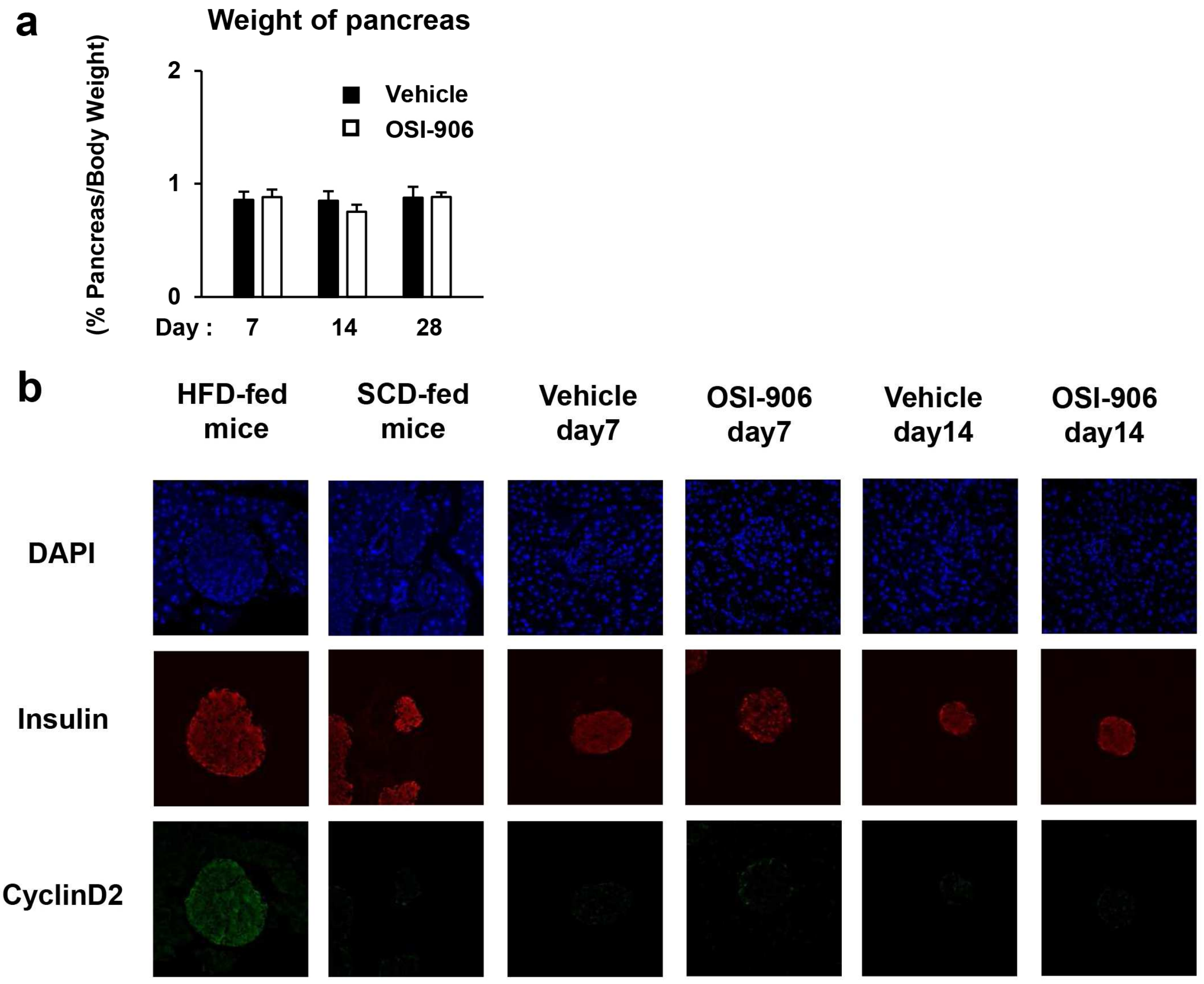

Supplementary Figure S3. Immunofluorecence of CyclinD2 was not upregulated by administration of OSI-906.

(a) Ratio of the pancreas weight to the body weight on days 7, 14, and 28. Data represent the mean ± SEM (n = 8 per group). (b) Sections of pancreas in C57BL/6J male mice fed a High-fat diet (HFD) (positive control) or Standard chow diet (SCD) for 8 weeks, and mice treated with Vehicle or OSI-906 on days 7 and 14. DAPI, Insulin, and Cyclin D2 are stained blue, red, and green, respectively. The sections were immunostained with antibodies to insulin (Santa Cruz) or CyclinD2 (Santa Cruz) and Alexa Fluor 488- and 647-conjugated secondary antibodies (Invitrogen) were used.

Supplementary Figure S4

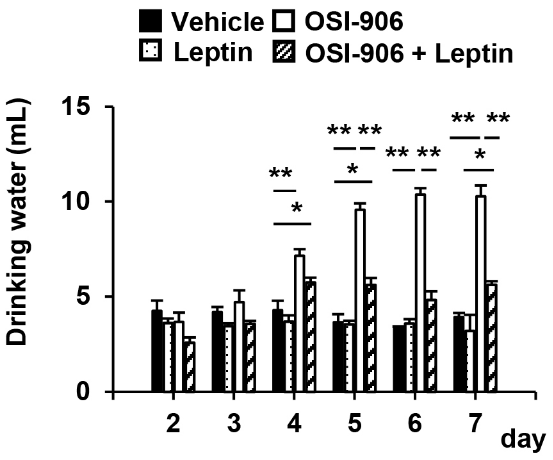

Supplementary Figure S4. Water consumption in OSI-906 and leptin treated mice.
Consumption of drinking water in indicated mice. Mice were housed 3-4 per cage. Consumption of drinking water were monitored per cage during a period of 7 days. Data represent the mean ± SEM. *$P \leq 0.05$, **$P \leq 0.01$.

Supplementary Figure S5

Fig.1a (left:Liver)

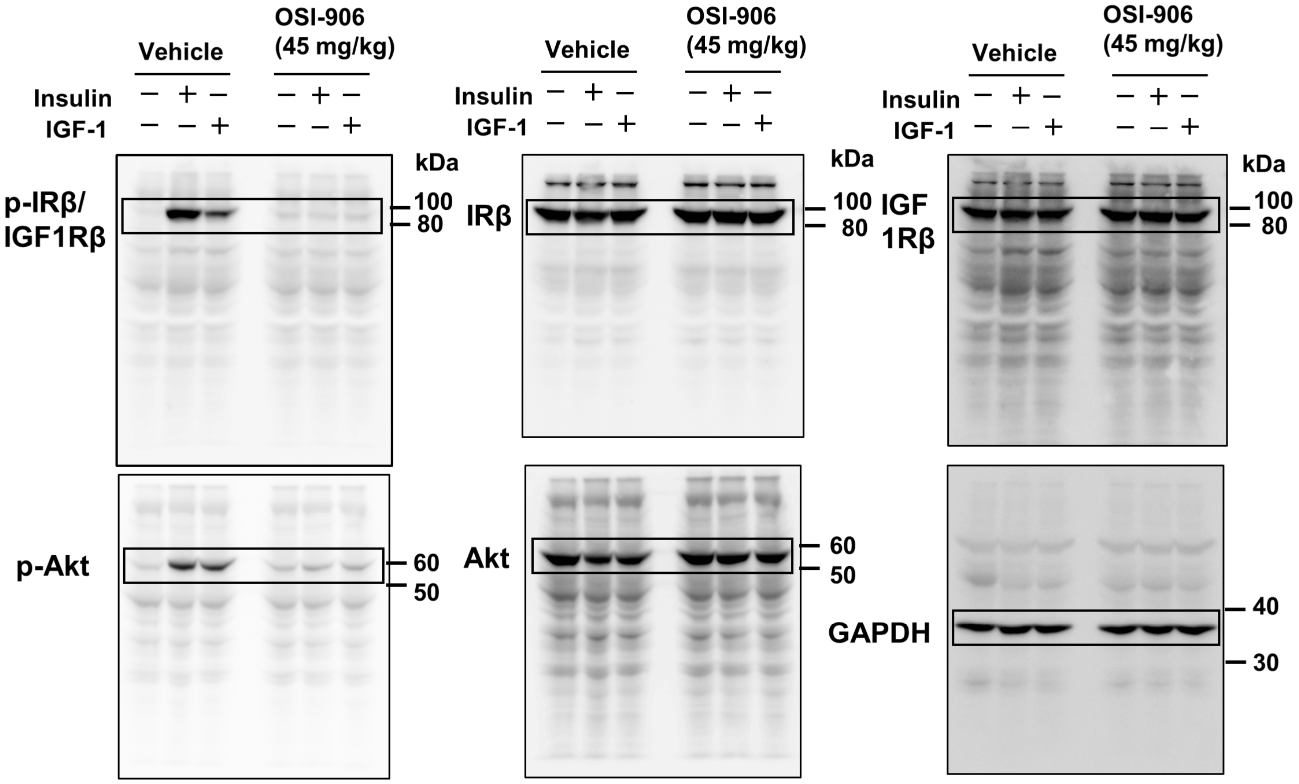

Fig.1a (right: Epididymal fat)

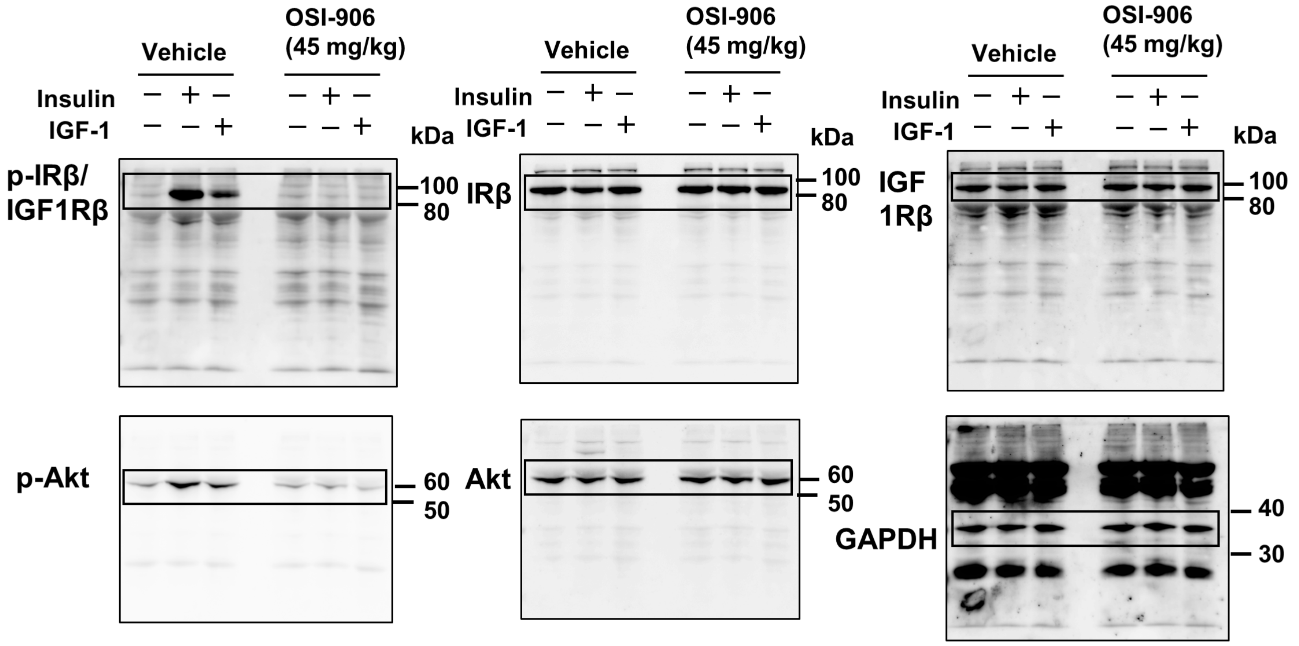

Supplementary Figure S5. Uncropped images of key panels in main figures.

Black boxes indicate the cropped portion of each immunoblot presented in the corresponding main figure.

Supplementary Table S1. Primer sequences.

Gene Name	FORWARD	REVERSE
Acc1	5’-ATGGGCGGAATGGTCTCTTTC-3’	5’-TGGGGACCTTGTCTTCATCAT-3’
Acc2	5’-CGCTCACCAACAGTAAGGTGG-3’	5’-GCTTGGCAGGGAGTTCCTC-3’
Aebp1	5’-TTGGAAACGCTGGATCGGTTA-3’	5’-CTTGACCTTGCCAGGCATTT-3’
Adipoq	5’-TGTTCCTCTTAATCCTGCCCA-3’	5’-CCAACCTGCACAAGTTCCCTT-3’
ANGPTL8	5’-CACTGTACGGAGACTACAAGTGC-3’	5’-GTGGCTCTGCTTATCAGCTCG-3’
aP2	5’-GCGTGGAATTCGATGAAATCA-3’	5’-CCCGCCATCTAGGGTTATGA-3’
Atf6	5’-TCGCCTTTTAGTCCGGTTCTT-3’	5’-GGCTCCATAGGTCTGACTCC-3’
Atgl	5’-CTTGAGCAGCTAGAACAATG-3’	5’-GGACACCTCAATAATGTTGGC-3’
BiP	5’-ACCTATTCCTGCGTCGGTGT-3’	5’-GCATCGAAGACCGTGTTCTC-3’
Ccl2	5’-TTAAAAACCTGGATCGGAACCAA-3’	5’-GCATTAGCTTCAGATTTACGGGT-3’
Ccna2	5’-GCCTTCACCATTCATGTGGAT-3’	5’-TTGCTGCGGGTAAAGAGACAG-3’
Ccnb1	5’-AAGGTGCCTGTGTGTGAACC-3’	5’-GTCAGCCCCATCATCTGCG-3’
Ccnb2	5’-GCCAAGAGCCATGTGACTATC-3’	5’-CAGAGCTGGTACTTTGGTGTTC-3’
Ccnd1	5’-GCGTACCCTGACACCAATCTC-3’	5’-CTCCTCTTCGCACTTCTGCTC-3’
Ccnd2	5’-GAGTGGGAACTGGTAGTGTTG-3’	5’-CGCACAGAGCGATGAAGGT-3’
Ccne1	5’-GTGGCTCCGACCTTTCAGTC-3’	5’-CACAGTCTTGTCAATCTTGGCA-3’
Ccne2	5’-GACTGGATGGTGCCTTTTGT-3’	5’-GACAGCTGCCCTCCTTTTCT-3’
Cdk1	5’-AGGTACTTACGGTGTGGTGTAT-3’	5’-CTCGCTTTCAAGTCTGATCTTCT-3’
Cdk2	5’-CCTGCTTATCAATGCAGAGGG-3’	5’-TGCGGGTCACCATTTCAGC-3’
Cdk4	5’-ATGGCTGCCACTCGATATGAA-3’	5’-TCCTCCATTAGGAACTCTCACAC-3’
Cd8	5’-CTCTGGCTGGTCTTCAGTATGA-3’	5’-TCTTTGCCGTATGGTTGGTTT-3’
Cd11b	5’-ATGGACGCTGATGGCAATACC-3’	5’-TCCCCATTCACGTCTCCCA-3’
Cd11c	5’-CTGGATAGCCTTTCTTCTGCTG-3’	5’-GCACACTGTGTCCGAACTC-3’
Cd34	5’-AAGGCTGGGTGAAGACCCTTA-3’	5’-TGAATGGCCGTTTCTGGAAGT-3’
Cd36	5’-CCTTAAAGGAATCCCCGTGT-3’	5’-TGCATTTGCCAATGTCTAGC-3’
Cd40	5’-TTGTTGACAGCGGTCCATCTA-3’	5’-CCATCGTGGAGGTACTGTTTG-3’
Cd68	5’-TGTCTGATCTTGCTAGGACCG-3’	5’-GAGAGTAACGGCCTTTTTGTGA-3’
Cebpa	5’-TCTGCGAGCACGAGACGTC-3’	5’-GCCAGGAACTCGTCGTTGAA-3’
Chop	5’-CTGGAAGCCTGGTATGAGGAT-3’	5’-CAGGGTCAAGAGTAGTGAAGGT-3’
Cpt1a	5’-CTCCGCCTGAGCCATGAAG-3’	5’-CACCAGTGATGATGCCATTCT-3’
Dgat1	5’-GAGATTGGTGGAATGCTGAG-3’	5’-GCATAGGCTTGTAGAAGTGTC-3’
Fas	5’-CCTGGATAGCATTCCGAACCT-3’	5’-AGCACATCTCGAAGGCTACACA-3’
F4/80	5’-CTTTGGCTATGGGCTTCCAGTC-3’	5’-GCAAGGAGGACAGAGTTTATCGTG-3’
Foxm1	5’-CAGAATGCCCCGAGTGAAACA-3’	5’-GTGGGGTGGTTGATAATCTTGAT-3’
Gck	5’-GCGGAGATGCTCTTTGAC-3’	5’-GTCCCACGATGTTGTTCC-3’

Glut4	5’-CCGCGGCCTCCTATGAGATACT-3’	5’-AGGCACCCCGAAGATGAGT-3’
Gys2	5’-CCAGCTTGACAAGTTCGACA-3’	5’-ATCAGGCTTCCTCTTCAGCA-3’
G6pase	5’-TCTGTCCCGGATCTACCTTG-3’	5’-GAAAGTTTCAGCCACAGCAA-3’
Hk2	5’-TGATCGCCTGCTTATTCACGG-3’	5’-AACCGCCTAGAAATCTCCAGA-3’
Hsl	5’-GCTGGAGGAGTGTTTTTTTGC-3’	5’-AGTTGAACCAAGCAGGTCACA-3’
Igfbp1	5’-ATCAGCCCATCCTGTGGAAC-3’	5’-TGCAGCTAATCTCTCTAGCACTT-3’
Igfbp2	5’-CAGACGCTACGCTGCTATCC-3’	5’-CCCTCAGAGTGGTCGTCATCA-3’
Il1b	5’-GGGCCTCAAAGGAAAGAATC-3’	5’-TTGCTTGGGATCCACACTCT-3’
Il6	5’-TAGTCCTTCCTACCCCAATTTCC-3’	5’-TTGGTCCTTAGCCACTCCTTC-3’
Il10	5’-GCTCTTACTGACTGGCATGAG-3’	5’-CGCAGCTCTAGGAGCATGTG-3’
Ire1	5’-ACACCGACCACCGTATCTCA-3’	5’-CTCAGGATAATGGTAGCCATGTC-3’
Ki67	5’-ATCATTGACCGCTCCTTTAGGT-3’	5’-GCTCGCCTTGATGGTTCCT-3’
Leptin	5’-GAGACCCCTGTGTCGGTTC-3’	5’-CTGCGTGTGTGAAATGTCATTG-3’
Lpl	5’-GGGAGTTTGGCTCCAGAGTTT-3’	5’-TGTGTCTTCAGGGGTCCTTAG-3’
Mgl	5’-TCGGAACAAGTCGGAGGT-3’	5’-TCAGCAGCTGTATGCCAAAG-3’
Mpo	5’- CCATGGTCCAGATCATCACA-3’	5’- CCATGGTCCAGATCATCACA-3’
p16	5’-CGCAGGTTCTTGGTCACTGT-3’	5’-TGTTCACGAAAGCCAGAGCG-3’
p21	5’-CCTGGTGAGTCCGACCTG-3’	5’-CCATGAGCGCATCGAATC-3’
p27	5’-TCAACGTGAGAGTGTCTACG-3’	5’-CCGGGCGAAGAGATTTCTG-3’
Pepck	5’-CTGCATAACGGTCTGGACTTC-3’	5’-CAGCAACTGCCCGTACTCC-3’
Perilipin	5’-TGCTGGATGGAGACCTC-3’	5’-ACCGGCTCCATGCTCCA-3’
Pgc1a	5’-AACCACACCCACAGGATCAGA-3’	5’-TCTTCGCTTTATTGCTCCATGA-3’
Ppara	5’-ACGATGCTGTCCTCCTTGATG-3’	5’-GTGTGATAAAGCCATTGCCGT-3’
Pparg	5’-ATGCCAAAAATATCCCTGGTTTC-3’	5’-GGAGGCCAGCATCGTGTAGA-3’
Pref-1	5’-AATAGACGTTCGGGCTTGCA-3’	5’-GGAGCATTCGTACTGGCCTTT-3’
Pygl	5’-GAGAAGCGACGGCAGATCAG-3’	5’-CTTGACCAGAGTGAAGTGCAG-3’
Scd-1	5’-CCGGAGACCCCTTAGATCGA-3’	5’-TAGCCTGTAAAAGATTTCTGCAAACC-3’
SerpinB1	5’-GCTGCTACAGGAGGCATTGC-3’	5’-CGGATGGTCCACTGTGAATTC-3’
Srebp1c	5’-GGCACTAAGTGCCCTCAACCT-3’	5’-GCCACATAGATCTCTGCCAGTGT-3’
Survivin	5’-GAGGCTGGCTTCATCCACTG-3’	5’-CTTTTTGCTTGTTGTTGGTCTCC-3’
Tnf	5’-CCCTCACACTCAGATCATCTTCT-3’	5’-GCTACGACGTGGGCTACAG-3’
β-actin	5’-CTAAGGCCAACCGTGAAAAGAT-3’	5’-CTAAGGCCAACCGTGAAAAGAT-3’
Tbp	5’-GGGGAGCTGTGATGTGAAGT-3’	5’-CCAGGAAATAATTCTGGCTCA-3’
